# Prize Essays

**Published:** 1853-01

**Authors:** 


### PRIZE ESSAYS.

It will be seen that the editors of the American Journal of Dental Science, have offered very liberal premiums for four prize essays. We hope this will bring out some of the talent of the profession. The subjects are all good and embrace sufficient to bring out those who are either fond of pathological, practical or mechanical study.

The conductors of this Journal certainly deserve much credit for their unwearied exertions in the cause of dental science; and we hope their valuable Journal will still continue to extend its circulation and usefulness every year, and ultimately repay them for all their labor.

